# Change in Vitamin D Levels Occurs Early after Antiretroviral Therapy Initiation and Depends on Treatment Regimen in Resource-Limited Settings

**DOI:** 10.1371/journal.pone.0095164

**Published:** 2014-04-21

**Authors:** Fiona P. Havers, Barbara Detrick, Sandra W. Cardoso, Sima Berendes, Javier R. Lama, Patcharaphan Sugandhavesa, Noluthando H. Mwelase, Thomas B. Campbell, Amita Gupta, for the ACTG A5175 PEARLS and NWCS319 Study Teams

**Affiliations:** 1 Johns Hopkins University School of Medicine, Baltimore, Maryland, United States of America; 2 Johns Hopkins University Bloomberg School of Public Health, Baltimore, Maryland, United States of America; 3 Evandro Chagas Clinical Research Institute (IPEC), FIOCRUZ, Rio de Janeiro, Brazil; 4 College of Medicine-Johns Hopkins University Research Project, Blantyre, Malawi; 5 Liverpool School of Tropical Medicine, Liverpool, United Kingdom; 6 Asociacion Civil Impacta Salud y Educacion, Lima, Peru; 7 Chiang Mai University, Chiang Mai, Thailand; 8 University of Witswatersrand, Johannesburg, South Africa; 9 University of Colorado Denver, Aurora, Colorado, United States of America; UCL Institute of Child Health, University College London, United Kingdom

## Abstract

**Study Background:**

Vitamin D has wide-ranging effects on the immune system, and studies suggest that low serum vitamin D levels are associated with worse clinical outcomes in HIV. Recent studies have identified an interaction between antiretrovirals used to treat HIV and reduced serum vitamin D levels, but these studies have been done in North American and European populations.

**Methods:**

Using a prospective cohort study design nested in a multinational clinical trial, we examined the effect of three combination antiretroviral (cART) regimens on serum vitamin D levels in 270 cART-naïve, HIV-infected adults in nine diverse countries, (Brazil, Haiti, Peru, Thailand, India, Malawi, South Africa, Zimbabwe and the United States). We evaluated the change between baseline serum vitamin D levels and vitamin D levels 24 and 48 weeks after cART initiation.

**Results:**

Serum vitamin D levels decreased significantly from baseline to 24 weeks among those randomized to efavirenz/lamivudine/zidovudine (mean change: −7.94 [95% Confidence Interval (CI) −10.42, −5.54] ng/ml) and efavirenz/emtricitabine/tenofovir-DF (mean change: −6.66 [95% CI −9.40, −3.92] ng/ml) when compared to those randomized to atazanavir/emtricitabine/didanosine-EC (mean change: −2.29 [95% CI –4.83, 0.25] ng/ml). Vitamin D levels did not change significantly between week 24 and 48. Other factors that significantly affected serum vitamin D change included country (p<0.001), season (p<0.001) and baseline vitamin D level (p<0.001).

**Conclusion:**

Efavirenz-containing cART regimens adversely affected vitamin D levels in patients from economically, geographically and racially diverse resource-limited settings. This effect was most pronounced early after cART initiation. Research is needed to define the role of Vitamin D supplementation in HIV care.

## Introduction

Vitamin D has well-known regulatory functions in calcium metabolism, but is also increasingly recognized as an immune modulator [1]. It regulates pathways involved in killing intracellular pathogens [2], and modulates T cells, cytokines, and dendritic cells [3]–[6]. Given these wide-ranging effects, vitamin D deficiency may lead to defects in both innate and adaptive immune defenses.

Inadequate vitamin D (defined here as serum 25(OH) vitamin D level <32 ng/ml) is widespread among HIV-infected persons, with prevalence estimates ranging from 29 to 89 percent in European and US HIV-infected populations [7]–[12]. Several studies have suggested that inadequate vitamin D levels are associated with worse clinical outcomes [9], [13]–[15]. Specific antiretroviral medications, including efavirenz and zidovudine, have been associated with lower serum vitamin D levels and may exacerbate this problem [16]–[20].

Studies examining the impact of antiretroviral medications on vitamin D have been done in North American and European HIV-infected populations. Genetics influence vitamin D levels, as do factors affecting UV light exposure and absorption, such as skin pigmentation, cultural practices, season and latitude [21]–[23]. In addition, more than 90% of HIV-infected people live in resource-limited settings (RLS), where the prevalence of vitamin deficiencies, including vitamin D deficiency, is high [24], [25]. Given the ongoing scale-up of antiretroviral therapy in RLS, understanding the impact of combination antiretroviral (cART) initiation on vitamin D levels in RLS is essential. We examined the effect of cART initiation with three different regimens in nine geographically, economically and racially diverse countries.

## Methods

### Study Population

The study population was a nested cohort of the Prospective Evaluation of Antiretrovirals in Resource Limited Settings (PEARLS) trial (Adult AIDS Clinical Trials Group (ACTG) A5175, NCT00084136), an open-label, randomized clinical trial of three cART regimens. The parent study population has been described in detail elsewhere [26], but in brief, participants were 1571 adults, 18 years or older from 9 countries (Brazil, Haiti, Peru, Thailand, India, Malawi, South Africa, Zimbabwe and the United States) on four continents, primarily RLS. They were HIV-infected with a CD4 count less than 300 cells/mm^3^, antiretroviral naïve, not pregnant and were excluded if they were acutely ill or had acute serious co-morbidities or laboratory abnormalities.

The study was approved by the institutional review boards and ethics committees at participating institutions, with written, informed consent obtained from all participants.

### Design

Subjects were randomly assigned 1∶1∶1 to one of three open-label regimens. Arm A was efavirenz 600 mg daily plus lamivudine-zidovudine 150 mg/300 mg twice daily; Arm B was atazanavir 400 mg, didanosine-EC 400 mg and emtricitabine 200 mg once daily; Arm C was efavirenz 600 mg plus emtricitabine-tenofovir-DF 200 mg/300 mg once daily. This sub-study used a pre-specified subcohort of 270 participants. Thirty participants, stratified by treatment arm, were randomly chosen from each country.

### Laboratory Methods

Serum specimens were collected before or at entry to the PEARLS study and were stored at −70 Celsius. Serum vitamin D levels were measured at a single Clinical Laboratory Improvement Amendments (CLIA)-certified laboratory at the Johns Hopkins Hospital using the chemiluminescence immunoassay (DiaSorin, Stillwater, MN), which measures both vitamin D2 and D3 and is reported as a total serum 25(OH) vitamin D level [27].

### Definitions and Measurement

Vitamin D insufficiency was defined as 25(OH) vitamin D <32 ng/ml and deficiency as <20 ng/ml [28]. Race was defined as black, white, Asian, other, or more than one race. Seasons were defined depending on the hemisphere of the country. For northern hemisphere countries, spring was defined as months 3–5 (southern hemisphere: months 9–11); summer, months 6–8 (southern hemisphere: months 12–2); autumn, months 9–11 (southern hemisphere: months 3–5), and winter, months 12–2 (southern hemisphere: months 6–8). Vitamin D levels were expected to be lower in winter and spring when compared to summer and autumn [8], [9], [29]. Subsequently, in this analysis, winter and spring were defined as “low vitamin D months” while the reciprocal months were defined as “high vitamin D months.” Normal weight range was defined as body mass index (BMI) 18–25 kg/m^2^, with overweight >25 kg/m^2^.

### Outcomes

**Figure 1:**
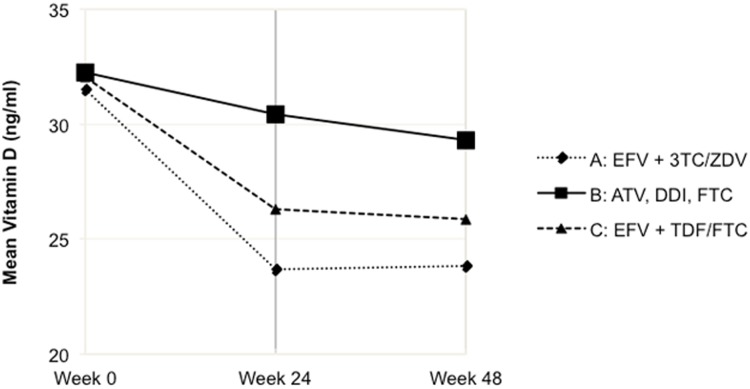
Mean Vitamin D Level by Treatment Group. **Abbreviations**: EFV, efavirenz; 3TC, lamivudine; ZDV, zidovudine; ATV, atazanavir; DDI, didanosine-EC; FTC, emtricitabine; TDF, tenofovir-DF.

The primary outcome was the change in serum 25-OH vitamin D level between baseline and 24 weeks. The change between week 24 and 48-week was also analyzed. However since analyses indicated that the largest change in Vitamin D occurred in the first 24 weeks post cART initiation **(** **Figure 1** **)**, predictor models described below only used the change between week 0 and week 24.

### Statistical Analysis

Background characteristics among treatment groups were assessed using Pearson’s chi-squared test. Mean change in vitamin D levels from cART initiation to 24 weeks after cART initiation and 95% confidence intervals, were estimated using linear regression models. First, univariate models were examined. This analysis was nested in a randomized controlled trial, which theoretically should control for measured and unmeasured confounder at baseline. However, some variation between groups in confounding factors will inevitably occur by chance, and a multivariate model that controls for additional confounders can also be used; it can increase the precision of the estimate of the association between exposure and outcome, as well as provide additional information about factors beyond treatment regimens that may affect the change in vitamin D level after cART initiation. In choosing covariates for the multivariate linear regression models, in addition to treatment group, all covariates that had been shown to influence baseline vitamin D levels in this study population [29] were initially included in the model. These included HIV viral load, race, country, season of baseline sample, and BMI. Sex, age, baseline vitamin D level, baseline CD4 count, baseline hemoglobin, baseline creatinine clearance, and baseline albumin level were also included in the multiple linear regression models because there was biologic plausibility of the effect of the variable on the change in vitamin D level or the covariate had face validity as a potential confounder. Models were evaluated using the Akaike information criterion (AIC) to determine the most parsimonious model.

In all analyses, intention-to-treat principle was followed. The a priori significance level was set at alpha = 0.05 for a two-sided significance test. Data were analyzed using Stata/IC version 12.1.

## Results

A total of 220 (81%) of 270 randomly selected persons had both week 0 and week 24 vitamin D measurements, 222 (82%) had week 0 and week 48, and 205 (76%) had weeks 0, 24, and 48. Fifty subjects, most from a study site in India, were excluded for either missing baseline or 24 week data. Besides country and race, those with missing data were significantly more likely to be underweight than other subjects, but otherwise did not differ significantly from the rest of the sample (data not shown). Four participants who died before 24 weeks were excluded.

Baseline age, sex, race, BMI, and baseline CD4 count, HIV viral load, and serum vitamin D levels (median 32 ng/ml; IQR 25–39 ng/ml) were similar between treatment arm groups (**Table 1**). 52% of the patients had serum vitamin D levels ≥32 ng/ml (adequate). In contrast, 48% of patients had serum vitamin D levels <32 ng/ml (insufficient) and 14% had <20 ng/ml (deficient).

**Table 1:** Study population characteristics by treatment arm.

Characteristic			Treatment Group			
		All	A: EFV+3TC/ZDV	B: ATV + DDI + FTC	C: EFV + TDF/FTC	p-value
All, no.		220	79	76	65	
Sex, no. (%)	Male	106 (48)	41 (52)	38 (50)	27 (42)	0.43
	Female	114 (52)	38 (48)	38 (50)	38 (58)	
Age–years, median (IQR)		35 (30–42)	35 (30–40)	35 (30–42)	34 (30–42)	0.88
Country, no. (%)	Brazil	29 (13)	12 (15)	9 (12)	8 (12)	0.58
	Haiti	27 (12)	11 (14)	11 (14)	5 (7)	
	India	5 (2)	2 (3)	2 (3)	1 (2)	
	Malawi	24 (11)	11 (14)	7 (9)	6 (9)	
	Peru	28 (13)	10 (13)	5 (7)	13 (20)	
	South Africa	26 (12)	8 (10)	10 (13)	6 (9)	
	Thailand	26 (12)	7 (9)	10 (13)	11 (16)	
	United States	27 (13)	6 (8)	10 (13)	6 (9)	
	Zimbabwe	28 (13)	12 (15)	10 (12)	7 (10)	
Race, no. (%)	White	27 (12)	5 (6)	13 (17)	9 (14)	0.46
	Black	127 (57)	53 (67)	44 (58)	30 (46)	
	Asian	32 (15)	11 (11)	12 (16)	11 (17)	
	Other*	33 (16)	12 (15)	7 (9)	14 (22)	
BMI, no. (%)	BMI <18.5 kg/m^2^	16 (7)	6 (8)	6 (8)	4 (6)	0.87
	BMI 18–25 kg/m^2^	143 (65)	55 (70)	46 (60)	42 (65)	
	BMI ≥25 kg/m^2^	61 (28)	18 (22)	24 (32)	19 (29)	
Prior AIDS diagnosis, no. (%)		16 (7)	6 (8)	6 (8)	4 (6)	0.92
Baseline CD4 count–cells/µl, median (IQR)		180 (87–231)	168 (64–245)	193 (126–241)	163 (82–216)	0.76
Baseline viral load–log_10_copies/ml, median (IQR)		5.07(4.61–5.48)	5.07 (4.63–5.48)	5.04 (4.55–5.46)	5.07 (4.64–5.57)	0.93
Baseline vitamin Dcategory, no. (%)	Severely deficient (≤10 ng/ml)	3 (1)	2 (3)	1 (1)	0 (0)	0.77
	Deficient (10–20 ng/ml)	28 (13)	8 (10)	9 (12)	11 (17)	
	Insufficient (20–32 ng/ml)	74 (34)	28 (35)	26 (34)	20 (31)	
	Adequate (≥32 ng/ml)	115 (52)	41 (52)	40 (53)	34 (52)	
Baseline vitamin D–ng/ml, median (IQR)		32 (25–39)	32 (25–39)	32 (25–39)	32 (25–43)	0.89

*The majority of those reporting race as “Other” were Mestizo from Peru, Brazil and Haiti. Abbreviations: EFV, efavirenz; 3TC, lamivudine; ZDV, zidovudine; ATV, atazanavir; DDI, didanosine-EC; FTC, emtricitabine; TDF, tenofovir-DF; IQR, interquartile range; BMI, body mass index (kg/m^2^).

### Change in Vitamin D Levels from Baseline to Week 24 and Week 48 Post cART Initiation

Vitamin D levels decreased after cART initiation, with the greatest decrease in the first 24 weeks (**Table 2** **,** **Figure 1**). In univariate analysis comparing change in vitamin D in treatment arms from 0 to 24 weeks, levels declined significantly in both efavirenz-based arms (Arm A, mean change −7.94 ng/ml (95% confidence interval (CI) −10.42 to −5.54); Arm C, mean change −6.66 ng/ml (95% CI −9.40 to −3.92). The level in Arm B, the atazanavir-based arm, also decreased between 0 and 24 weeks, but this was not significant (mean change −2.30 ng/ml; 95% CI −4.86 to 0.25). There was no difference between the change in Arms A and C (p = 0.498), though arms A and C both differed from Arm B (p = 0.002 and p = 0.022 respectively). There was no significant change between overall mean Vitamin D levels at week 24 and week 48 (p = 0.34). There was also no significant between the specific Arms (Arm A vs Arm B, p = 0.24; Arm B vs C, p = 0.59; and Arm A vs C, p = 0.62); Arm A, mean change −0.22 ng/ml (95% CI −2.41 to −1.96); Arm B, mean change 1.52 ng/ml (95% CI −0.47 to 3.51); Arm C, mean change 0.63 ng/ml (95% CI −2.06 to 3.32) (**Table 2**).

**Table 2:** Mean change by treatment arm in serum vitamin D, 0 to 24 weeks and 24 to 48 weeks.

Mean change, Vitamin D ng/ml (CI)	A: EFV+3TC/ZDV	B: ATV+DDI +FTC	C: EFV+TDF/FTC	A = Bp-value	B = Cp-value	A = Cp-value
0 to 24 week (n = 220)	–7.9 (–10.4, −5.5)	–2.3 (–4.8, 0.3)	–6.7 (–9.4, −3.9)	0.002	0.022	0.50
24 to 48 week (n = 211)	–0.22 (–2.4, 2.0)	1.5 (–0.5, 3.5)	0.63 (–2.1, 3.3)	0.24	0.59	0.62

Abbreviations: EFV, efavirenz; 3TC, lamivudine; ZDV, zidovudine; ATV, atazanavir; DDI, didanosine-EC; FTC, emtricitabine; TDF, tenofovir-DF; IQR, interquartile range; BMI, body mass index (kg/m^2^).

### Independent Factors Associated with Change in Vitamin D Levels between Week 0 and Week 24

Multivariate analysis revealed that besides treatment arm, only country (p<0.001), baseline vitamin D (p<0.001), and season of baseline sample (p<0.001) were significantly associated with change in vitamin D level between week 0 and week 24. After adjusting for these factors, the relationship between treatment arms A and B (p<0.001) and between C and B was unchanged (p = 0.023).

In the initial model (Model A, **Table 3**), which was the saturated model with all possible confounders, country, baseline vitamin D and season of baseline sample, and treatment arm were the only covariates with a significant association with change in vitamin D level. There was strong co-linearity between race and country, and based on results from Model A, country appeared to have a stronger relationship with change in vitamin D compared with race. Although both variables predicted baseline vitamin D status, when examining change in vitamin D level, race had no significant association once controlling for country (Wald p = 0.34) (Model A, **Table 3**). Race was thus excluded from Model B (**Table 3**), as were baseline hemoglobin, albumin and creatinine clearance; in analyses in which these three latter variables were evaluated separately and together, none appeared to contribute to the model (Wald test p = 0.25). In Model B, after adjusting for potential factors that potentially affect change in vitamin D level (baseline vitamin D, age, sex, CD4, viral load, country, season BMI), the relationship between the effect of the treatment arms was similar. It also did not change substantially in the most parsimonious model by AIC (**Model C,** **Table 3**), which excluded all variables except treatment arm, baseline season, baseline vitamin D level and country.

**Table 3:** Results of regression analyses for change in vitamin D from 0 to 24 weeks*.

		Multivariate Model A		Multivariate Model B		Multivariate Model C	
Variable		β (95% CI)	*p-*value	β (95% CI)	*p-*value	β (95% CI)	*p-*value
Treatment Arm B^1^		REF		REF		REF	
Treatment Arm A^1^		–5.84 (–8.38, −3.30)	<0.01	–5.72 (–8.15, −3.28)	<0.01	–5.77 (–8.18, −3.37)	<0.01
Treatment Arm C^1^		–2.70 (–5.39, −0.006)	0.049	–3.00 (–5.58, −0.42)	0.02	–2.96 (–5.49, −0.42)	0.023
Baseline vit D (ng/ml)		–0.60 (–0.72, −0.48)	<0.01	–0.60 (–0.71, −0.49)	<0.01	–0.58 (–0.68, −0.47)	<0.01
Season^2^		–6.38 (–8.63, −4.14)	<0.01	–6.79 (–8.97, −4.60)	<0.01	–6.86 (–8.99, −4.73)	<0.01
**Country** 3			<0.01		<0.01		<0.01
	Brazil	REF		REF		REF	
	Haiti	–1.56 (–6.72–3.60)	0.55	–2.98 (–7.14–1.18)	0.16	–2.85 (–6.79, 1.10)	0.16
	India	–1.11 (–18.3, 16.1)	0.90	–7.81 (–15.4, −0.26)	0.043	–6.91 (–14.3, 0.44)	0.065
	Malawi	4.03 (–1.82, 9.87)	0.18	3.21 (–1.38, 7.80)	0.17	2.85 (–1.24, 6.94)	0.17
	Peru	–0.75 (–7.83, 6.33)	0.84	–4.82 (–9.03, −0.61)	0.025	–5.66 (–9.60, −1.71)	<0.01
	South Africa	–0.27 (–5.52, 4.97)	0.92	–0.87 (–5.12, – 3.39)	0.69	–1.19 (–5.26, 2.88)	0.56
	Thailand	4.75 (–11.6, 21.1)	0.57	–1.11 (–5.68, 3.47)	0.63	–1.61 (–5.80, 2.57)	0.45
	United States	–4.47 (–9.32, 0.38)	0.07	–4.18 (–8.39, 0.023)	0.051	–4.43 (–8.58, −0.27)	0.037
	Zimbabwe	6.62 (1.59–11.7)	0.01	6.17 (2.02, 10.3)	<0.01	6.24 (2.31, 10.2)	<0.01
BMI (kg/m^2^)		–0.25 (–0.57, 0.061)	0.11	–0.14 (–0.39, – 0.12)	0.30		
Age (year)		0.10 (–0.057, 0.26)	0.21	0.073 (–0.061, 0.21)	0.29		
Sex^4^		–0.53 (–3.37, 2.31)	0.71	0.34 (–1.95, 2.63)	0.77		
Baseline CD4 (cells/µL)		0.01 (–0.01, 0.024)	0.24	0.007 (–0.01, 0.021)	0.34		
Viral load (log_10_VL)		0.33 (–1.35, – 2.01)	0.70	0.61 (–0.99, 2.20)	0.46		
History of AIDS^5^		–2.17 (–6.74, 2.41)	0.35				
Hemoglobin (g/dL)		0.43 (–0.34, 1.21)	0.27				
Creatinine Clearance (ml/min)		0.039 (–0.019, 0.10)	0.18				
Albumin (g/dL)		–1.62 (–4.00, 0.75)	0.18				
**Race** 6			0.34				
	Asian	REF					
	Black	5.11 (–10.6, 20.8)	0.52				
	White	6.62 (–9.04, 22.3)	0.41				
	Other	0.95 (–16.0, 17.9)	0.91				
Constant		13.3 (–11.3, 38.0)	0.29	17.1 (3.26, 30.9)	0.02	20.3 (15.5, 25.4)	<0.001
*R* 2		0.62		0.60		0.60	
*N*		216		220		220	

*Abbreviations: CI, confidence interval; BMI, body mass index; β values are unstandardized regression coefficients.

1 Both treatment arms A and C are compared to treatment arm B.

2 Reference group is summer/fall (“high vitamin D season”), defined as months 12–5 (southern hemisphere) or 6–11 (northern hemisphere). Comparison group is reciprocal seasons for each hemisphere respectively.

3 Wald p-value for overall p-value for country in multivariate analyses. For individual countries in multivariate analayses, p-value is compared to reference country Brazil.

4 Reference group is female.

5 Reference group has no self-reported history of AIDS prior to study entry.

6 Wald p-value for overall p-value for race. For individual countries in multivariate analyses, p-value is compared to reference race Asian.

When the analyses were repeated with percent change from baseline vitamin D level to 24-week vitamin D level, rather than absolute change in vitamin D level, there were no substantive differences in the results. Those with the highest baseline vitamin D levels showed the greatest percent decrease, and the effect of treatment regimen on change in vitamin D was similar when percent change was examined rather than absolute change (data not shown).

**Figure 2:**
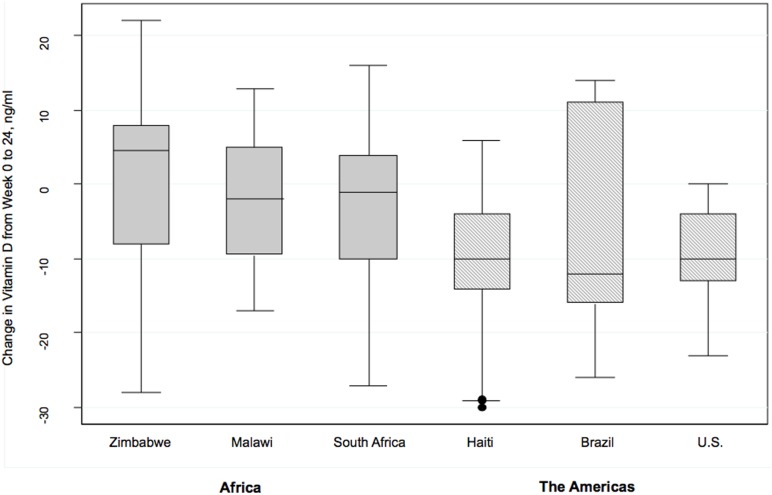
Change in vitamin D level from 0 to 24 weeks in blacks by country*. *25-OH Vitamin D level measured in ng/ml. Results are from multivariate linear regression analysis controlling for treatment arm, season, baseline 25-OH vitamin D level, CD4, viral load, age, sex and body mass index.

Country did affect the change in vitamin D level (Wald p<0.001). Declines in vitamin D level were statistically significantly greater in India and Peru when compared with the reference country Brazil (p = 0.043 and p = 0.025 respectively), with those in Zimbabwe having a relative increase in vitamin D level when compared to Brazil (p = 0.004). Because of the complex interaction of race and geography, and previous findings that differences in skin pigmentation and genetic polymorphisms associated with specific racial groups affect vitamin D metabolism, further sensitivity analyses of racial groups was performed. Blacks in the Americas (United States, Haiti and Brazil) had a greater decline in vitamin D level when compared to blacks from Africa (Zimbabwe, Malawi, and South Africa) (difference in mean change −5.1 ng/ml; 95% CI −8.3 to −1.9) (**Figure 2**). When US blacks were compared to all non-US blacks, there was a larger decrease in vitamin D level at 24 weeks (difference in mean change −9.42; 95% CI −15.2 to −3.6) ng/ml). There was no significant interaction between treatment arm and any racial group.

## Discussion

Our study demonstrated that serum vitamin D levels decline significantly in the first 24 weeks of initiating two different efavirenz-based regimens, but not an atazanavir-based regimen. Furthermore, after this initial change, serum vitamin D levels did not change significantly between week 24 and week 48, indicating that most of the effect on vitamin D occurs soon after antiretroviral treatment initiation. These findings were consistent in treatment-naïve patients across diverse racial and geographic settings, including resource-limited settings.

A number of previous studies have shown an association between efavirenz and increased risk of vitamin D deficiency, although most have been done in North American or European settings [17], [19], [20], [30]. These include a French cohort study that showed that efavirenz, but not other antiretroviral medications, was associated with vitamin D deficiency [30]. Another study among 87 HIV patients in the United States who had baseline vitamin D levels measured prior to antiretroviral initiation and again 6 to 12 months later found that those given efavirenz-containing regimen had a significantly greater drop than the non-efavirenz group [20]; a Swiss cohort found that although cART exposure in general had no significant impact on vitamin D levels, NNRTI use was associated with lower levels [8]. These findings were confirmed by a cross-sectional study conducted in London, where efavirenz use was found to be associated with severe vitamin D deficiency (<10 ng/ml) [19]. Although efavirenz has been the antiretroviral drug most implicated in lowering vitamin D levels, cART regimens contain multiple medications, and it is possible that the other medications besides efavirenz had an effect on vitamin D. Both efavirenz and zidovudine were associated with vitamin D deficiency in the MONET trial, which was conducted among European HIV-positive adults on cART with HIV RNA <50 copies/ml, on a wide variety of regimens at baseline. In addition, when study participants were changed to a regimen with boosted darunavir, vitamin D levels rose, with the greatest increase in vitamin D levels was among those who changed from efavirenz or zidovudine [17]. However, in our study, zidovudine did not appear to be significantly associated with change in vitamin D level, as there was no significant difference between the arms that contained efavirenz plus lamivudine-zidovudine when compared to efavirenz plus emtricitabine-tenofovir-DF.

The mechanism of efavirenz’s effect on vitamin D has been linked to induction of cytochrome P450, and the effect this has on several enzymes in this family. These include CYP24, which converts both 25(OH) vitamin D and the active form of vitamin D, 1,25(OH)_2_ vitamin D, to their inactive metabolites. Efavirenz also reduces the expression of cytochrome CYP2R1, which hydroxylates D3 and D2, necessary for vitamin D activation [31], [32]. However, it is unclear why vitamin D levels appear to drop most sharply in the first six months after cART initiation, but then appear to have a much smaller decline, if any, between 24 and 48 weeks. We postulate that there may be an initial induction of cytochrome P450 enzymes by efavirenz and then stabilization after maximal effect, and this occurs most prominently within the first 24 weeks. Alternatively it is possible that while there are early changes in the vitamin D protein binding [33] or regulation after cART initiation, compensatory mechanisms stabilize vitamin D levels over the long term, although this has yet to be clearly elucidated.

A number of factors have been shown to affect baseline vitamin D levels, such as BMI, non-white race, viral load, season of sample, and country [11], [18], [29]. Geographical factors such as latitude and altitude, as well as environmental factors such air pollution, can also affect vitamin D levels; cultural practices such avoiding sun exposure can also have an impact [23]. The relatively high median vitamin D values in this study population compared to other cohorts of HIV-infected individuals likely reflects the geographic, economic, racial and cultural diversity of this study population. In addition, we chose to include both deficiency and insufficiency together using the recommended pre-specified cut-off of <32 ng/ml for the Diasorin assay because, although severe vitamin D deficiency was relatively uncommon in our study, vitamin D insufficiency was relatively common in all country-race groups and has widespread clinical relevance [28].

In this study, those who started with the highest vitamin D levels had the greatest declines after cART initiation, examined both by absolute change in vitamin D levels and percent change from baseline to 24 weeks. However, once baseline vitamin D level is controlled for, most of these other factors did not appear to influence the decrease in vitamin D that occurs after cART initiation. Country and season of sample were the exceptions in this analysis. Country appeared to have a significant impact on the change in vitamin D, although a clear pattern was not readily apparent. A complex pattern of factors such as nutrition, racial composition, latitude, elevation, and culture potentially affect the role that country plays on change in vitamin D after cART initiation. Seasonality also affects vitamin D status; those with baseline samples drawn in summer and autumn (“high vitamin D” seasons), and thus with 24-week samples drawn in fall or winter (“low vitamin D” seasons), experienced a greater drop in the six months after cART initiation than those with a reciprocal change from “low” to “high” vitamin D seasons. However, the effect of treatment arm on change in vitamin D remained robust after controlling for season and country.

Our study had some limitations. Subjects were excluded if they had any acute illness or major lab abnormality. Thus, the clinical trial population may be healthier than patients in routine HIV care programs in comparable RLS. Our study was limited to three specific anti-retroviral regimens. However, atazanavir and efavirenz-containing combinations are commonly used globally. In addition, we did not have complete documentation on use of vitamin D supplementation; however vitamin D supplementation during the study conduct was reportedly very low in RLS. Patients were excluded from analysis if they were missing either baseline or 24-week vitamin D data. However, apart from country, race, and body mass index, the excluded patients did not significantly differ from the analytic sample, and the missing data would not be expected to bias our study results. In addition, we examined the results based on an intention-to-treat model. A limited number of patients switched regimens or discontinued cART after initiation [26], but we did not have enough data or a large enough sample size with vitamin D samples to exam the effect of stopping antiretroviral medications on vitamin D levels.

## Conclusions

Patients initiating two efavirenz-based cART regimens had a significant early decline in their vitamin D levels when compared to those initiating an atazanavir-based regimen. Further studies are needed to explore whether early cART-related declines in vitamin D level are clinically significant, what the long-term effects may be, and whether vitamin D supplementation has any role.
